# An exploratory study of perceived and actual similarity in extraversion and moral attitudes toward manners in young adult friendships: Evidence from Japanese female students

**DOI:** 10.1186/s13104-026-07810-w

**Published:** 2026-04-10

**Authors:** Hiroshi Nitta

**Affiliations:** 1 Graduate School of Letters, Kyoto University, Yoshida Honmachi Sakyo-ku, Kyoto-shi, Kyoto 606-8501 Japan; 2 Graduate School of Human-Environment Studies, Kyushu University, 744 Motooka, Nishi-ku, Fukuoka-shi, Fukuoka 819-0395 Japan

**Keywords:** Friendships, Moral attitude, Personality, Similarity

## Abstract

**Objective:**

Humans form and maintain long-term cooperative relationships, such as friendships, regardless of genetic connections. Evidence indicates that similarities between individuals play a key role in social selection and preferences. This study examined whether friends show similarity in personality traits, moral attitudes, and conformity, and whether subjective similarity correlates with objective similarity based on questionnaire ratings. Japanese female college students (*N* = 30 pairs) in friendship relationships participated in a face-to-face questionnaire survey as a friend-pair group, and anonymous female participants (*N* = 30 pairs) completed an online survey as a random-pair group.

**Results:**

Permutation analyses showed non-significant group differences in similarity in all variables between the friend-pair and random-pair groups (all *p*s > 0.050). In the friend-pair group, however, associations between subjective and objective similarity were observed: greater perceived similarity in personality was significantly associated with smaller within-dyad differences in Extraversion (*ρ* = − 0.47, df = 28, *p* = 0.009) and moral attitudes towards manners (*p* = − 0.47, df = 28, *p* = 0.008). These correlational findings, derived from Japanese female college students, suggest a potential association between subjective similarity in personality and moral attitude among friends, while acknowledging possible effects of differing data collection modes.

**Supplementary Information:**

The online version contains supplementary material available at 10.1186/s13104-026-07810-w.

## Introduction

Friendship is a distinctive social relationship in which individuals expect close and reciprocal bonds with non-kin others [1, 2]. Friends support each other not only with material resources (e.g., food, money), but also with non-material resources such as advice and emotional support [3].

Young adulthood is a period when individuals actively form and maintain friendships [4]. Empirical evidence shows that young adults draw clearer distinction between friends and non-friends in altruistic judgments [5] and neuroscientific studies indicate that interactions between friends engage reward-related brain regions more strongly than interactions with strangers or being alone [6]. Wagner et al. [7] also showed that the areas are more activated when they believe their friends are sharing an emotion, compared to when they are alone.

An important question is how friendships are sustained over time despite genetic unrelatedness. Perceived similarity is one key mechanism that fosters interpersonal attraction and cohesion [8, 9]. Previous studies suggested that friends tend to resemble each other in personality characteristics [10–12] and that perceived similarity enhances relationship intensity and satisfaction [13]. However, similarity processes can also arise through broader social influences such as complementarity [14] and network homophily [15], suggesting that similarity within friendships is dynamically embedded in broader social and contextual structures, rather than emerging solely from a static structure of preferences.

Beyond personality, moral attitudes—defined here as individuals’ evaluations of behaviours in relation to fairness, harm, or social norms [16, 17] —are central to cooperative and cohesive social life. Moral attitudes may be also shaped dynamically through social attributions, group membership, and cultural expectations [18–20]. In addition, previous work suggested that friends tend to resemble one another in moral attitudes [21] and conformity or value similarity [22]. Although research on moral reasoning [23–25] and shared values [26, 27] has been extensively studied, the role of concrete moral attitudes within friendships remains underexplored. Furthermore, it remains unclear whether friends’ subjective perceptions of psychological similarity correspond to their similarity in moral attitudes or conformity.

The present study addresses these gaps by examining whether similarities in personality traits, moral attitudes, and conformity are evident among friends, and whether perceived similarity corresponds to objectively measured similarity. The sample of Japanese female college students was selected because women typically form more intimate and emotionally expressive friendships than men [28, 29], and Japanese cultural norms emphasise interdependence and social harmony [30]. Among Japanese young women, emotional support from friends is strongly associated with psychological well-being, including gratitude and a sense of coherence [31]. Recent research further suggests that East Asian morality prioritises interpersonal obligations and relational harmony [32, 33], and that cultural contexts influence how moral norms regulate social behaviour and peer relations [20, 34]. These perspectives provide a relevant context for examining friendship similarity in Japan.

Based on this literature, I propose two hypotheses. First, friends were expected to resemble each other more closely than randomly paired individuals in personality traits, moral attitudes, and conformity. Second, greater perceived similarity between friends was expected to be associated with greater objectively measured similarity in these characteristics. Perceived similarity between self and others may reinforce one’s own attitudes and feelings, thereby eliciting affective responses that enhance friendship bonds [13, 35].

## Methods

### Participan

This study involved 30 pairs of female college students and their female friends (friend-pair group; *N* = 60, *M*_age_ = 19.02 years, *SD* = 0.72 years, age range = 18–20 years) and 30 randomly formed pairs (random-pair group; *N *= 60, *M*_age_ = 19.42 years, *SD* = 0.67 years, age range = 18–20 years). A pairwise sample of 30–40 dyads is considered adequate for dyadic research [36–39]. In the friend-pair group, participants were eligible if they had been friends for at least six months and met at least three times a week. For the random-pair group, 60 undergraduate students of the same age were recruited through Cross Marketing, Inc. (Tokyo, Japan), and 30 anonymous pairs were generated randomly.

The study protocol and data use policy were disclosed on the recruitment page and at the beginning of the questionnaire. Participation proceeded only after informed consent was obtained. All participants received monetary incentives.

### Procedure

In the friend-pair group, the two friends participated simultaneously in a room at a women’s junior college in Japan. A partition was erected to prevent visual contact and potential influence. Paper questionnaires were distributed, and participants circled their responses. For the random-pair group, an online questionnaire was administered using the QIQUMO (Cross Marketing, Inc.). To enhance data quality, participants who gave identical ratings across all items (i.e., *SD* = 0) were to be excluded; however, no such cases were found in either group.

### Measures

The questionnaire consisted of five sections: demographic data (Q1), Big Five traits (Q2), moral attitudes (Q3), conformity (Q4), and perceived similarity with friends (Q5). The structure for the random-pair group was identical except that participants in the random-pair group did not answer the subjective similarity items (i.e., Q5). Since subjective similarity was assessed with a single global item asking how similar participants perceived themselves to be to their friend in personality and mindset, internal consistency coefficients (Cronbach’s α) were computed only for the other scales (i.e., Q1–Q4) (see Supplementary Information). In this study, mindset refers to a general way of thinking that shapes how individuals perceive and respond to various situations [40]; in Japanese, this was expressed as kangaekata (考え方).

All items for the Big Five traits, moral attitudes, conformity and subjective similarity are described in the Supplementary Information. Similarity between dyad individuals was operationalised using the absolute difference between their scores. Although this approach does not retain directional information, order within pairs was not the focus of the present study, and absolute differences are widely used as indices of similarity in dyadic analyses [39, 41].

### Personality

Personality traits were assessed using a short form of the Japanese Big Five personality traits [42], which includes Extraversion, Agreeableness, Conscientiousness, Neuroticism, and Openness to Experience. Items were rated on a 7-point scale (1 = “not at all applicable” to 7 = “very applicable”).

### Moral attitude

A 15-item Japanese questionnaire on moral and legal consciousness [43] was used to measure tolerance of immoral and illegal behaviours. Five items each assessed attitudes toward legal violations, ordinance violations, and violations of manners, rated on a 5-point scale (1 = “not at all allowed” to 5 = “very allowed”).

### Conformity

Conformity was assessed using a Japanese version of the conformity scale by Yokota and Nakanishi [44] It comprises 10 items on informational influence-based conformity (“informational conformity”) and 13 items regarding normative influence-based conformity (“normative conformity”), rated on a 5-point scale (1 = “disagree” to 5 = “agree”).

### Subjective similarity with friend

Participants in the friend-pair group rated subjective similarity in personality and mindset on a 5-point scale (1 = “not at all similar” to 5 = “extremely similar”).

**Table 1:** Means and standard deviations (SD) of similarity scores for personality traits, moral attitudes, and conformity in the friend-pair and random-pair groups

Variable		Friend-pair group (N = 30 pair)		Random-pair group (N = 30 pair)	
		Mean	SD	Mean	SD
Big Five personality traits	Extraversion	0.18	(0.10)	0.18	(0.11)
	Agreeableness	0.16	(0.12)	0.12	(0.09)
	Conscientiousness	0.14	(0.09)	0.15	(0.11)
	Neuroticism	0.21	(0.16)	0.21	(0.16)
	Openness to experience	0.15	(0.13)	0.13	(0.09)
Moral attitudes	Legal violations	0.10	(0.10)	0.21	(0.16)
	Ordinance violations	0.10	(0.08)	0.18	(0.16)
	Violations of manners	0.13	(0.10)	0.22	(0.15)
Conformity	Informational	0.10	(0.09)	0.12	(0.09)
	Normative	0.15	(0.11)	0.15	(0.12)

Similarity scores are based on absolute within-dyad differences. Lower scores indicate greater similarity in the variable between pairs. The absolute differences were obtained by computing the absolute value of the score difference between the two individuals in each dyad (i.e., |score₁ − score₂|), where smaller values represented higher within-dyad similarity

## Results

Table 1 presents descriptive statistics for the Big Five traits, moral attitudes, and conformity in the friend-pair and random-pair groups. All analyses were conducted in R version 4.2.2 [45]. Within-dyad similarity was indexed by the absolute difference between two individuals in each dyad (i.e., |score₁ − score₂|), with smaller values indicated greater similarity. These raw differences were standardised by dividing them by the maximum possible score. All supplementary correlational analyses and corresponding tables are reported in the Supplementary Material (see Supplemental Tables 2–4).

I tested whether (a) the mean absolute differences of each variable between pairs differed significantly between the friend-pair and random-pair groups, and (b) the associations between subjective similarity and objective variable differences were reliable, by using a non-parametric, distribution-free approach. To address potential incompatibility between the friend and random-pair samples, I conducted permutation tests [46, 47].

In the permutation tests, the observed statistic was the mean absolute difference between paired participants for each variable (e.g., Extraversion, Agreeableness, legal violation). The null hypothesis assumed that any observed difference could be attributed to random pairing of individuals. For each of 10,000 iterations (B = 10,000), I randomly permuted the participant ids and reformed pseudo-pairs, calculating the mean absolute differences for each variable at each iteration. Empirical *p*-values were obtained as the proportion of permuted statistics equal to or more extreme than the observed value (two-sided test).

The results for group differences showed that all observed mean absolute differences between friend and random pairs fell within the 95% permutation-based confidence intervals, indicating that the apparent group differences in similarity were not statistically distinguishable from random variation (all *p*s > 0.050 and see Supplemental Table 1).

Next the permutation test was conducted to examine associations between subjective similarity and objective similarity in the friend-pair group (see Table 2). Subjective similarity was computed as the mean of the two friends’ responses (*M*_Personality_ = 3.07, *SD*_Personality_ = 1.33; *M*_Mindset_ = 3.25, *SD*_Mindset_ = 1.16). Objective similarity was indexed by the absolute within-dyad difference score for each variable.

**Table 2:** Results of permutation tests for correlations between subjective (personality and mindset) and objective similarity across variables (*N* = 30 dyads)

		Variable	Observed *ρ*	*p* (permutation test)	CI_lower_	CI_upper_
Personality	Big Five personality traits	Extraversion	− 0.47	0.009	− 0.36	0.36
		Agreeableness	0.14	0.471	− 0.36	0.35
		Conscientiousness	0.09	0.629	− 0.35	0.37
		Neuroticism	0.14	0.450	− 0.36	0.37
		Openness to experience	0.10	0.577	− 0.36	0.36
	Moral attitudes	Legal	− 0.14	0.452	− 0.36	0.37
		Ordinance	− 0.09	0.633	− 0.36	0.36
		Manner	− 0.47	0.008	− 0.36	0.37
	Conformity	Informational	− 0.13	0.484	− 0.37	0.36
		Normative	− 0.23	0.216	− 0.37	0.36
Mindset	Big Five personality traits	Extraversion	− 0.23	0.227	− 0.37	0.36
		Agreeableness	0.00	0.996	− 0.36	0.36
		Conscientiousness	0.18	0.337	− 0.36	0.36
		Neuroticism	− 0.10	0.604	− 0.36	0.37
		Openness to Experience	0.02	0.914	− 0.36	0.36
	Moral attitudes	Legal	− 0.10	0.592	− 0.35	0.36
		Ordinance	0.04	0.837	− 0.37	0.36
		Manner	− 0.18	0.339	− 0.36	0.36
	Conformity	Informational	0.22	0.241	− 0.37	0.37
		Normative	0.35	0.061	− 0.37	0.36

Observed ρ denotes the Spearman’s rank correlation between perceived similarity (personality and mindset) and objectively measured variables. *p*-values were obtained using two-tailed permutation tests (10,000 iterations). CI_lower_ and CI_upper_ indicate the 2.5th and 97.5th percentiles of the null distribution of *ρ*, respectively

The results showed a significant negative association between subjective similarity in personality and differences in Extraversion (*ρ* = − 0.47, df = 28, *p* = 0.009) and moral attitudes toward violations of manners (*ρ* = − 0.47, df = 28, *p* = 0.008), indicating that higher perceived similarity was associated with smaller discrepancies in these domains. No other associations reached significance after permutation testing (*p*s > 0.061). All non-significant results from both the between-group comparisons and the associations between subjective and objective similarity are reported in the Supplementary Information.

## Discussion

This study examined whether friends in young adulthood show similarity in personality traits, moral attitudes, and conformity, and whether perceived similarity aligned with objectively measured similarity. Our permutation analyses indicated that non-significant group differences in similarity in all variables between the friend-pair and random-pair groups. However, subjective similarity in personality showed significant associations with objectively measured similarity in Extraversion and moral attitudes toward violations of manners in the friend-pair group.

One possible explanation is that the association between perceived similarity in personality and Extraversion reflects both the perceptual salience and social function of this trait. First, Extraversion is highly observable through behavioural cues (e.g., sociability, enthusiasm), making similarity relatively easy to detect [48, 49]. Extraverted behaviour also facilitates social engagement and relationship initiation [50], increasing opportunities for similarly extraverted individuals to form friendships. Importantly, this pattern does not imply that Extraversion plays a causal role in forming friendships, but suggests that its salience and social relevance may make it a key dimension for perceived similarity.

The association between perceived personality similarity and similarity in moral attitudes toward manners also offers insight into the social function of moral norms. Previous work suggests that moral norms shape expectations about social harmony and compatibility [51–53]. From adolescence through young adulthood, peer approval and social coordination become especially salient [54], although the present study focused specifically on young adulthood rather than adolescence. Agreement about socially appropriate behaviour—especially everyday manners—may therefore tentatively suggest that some domains of moral attitudes, such as manners, may play a more prominent role than others in shaping perceived similarity within friendships.

### Limitation

Several limitations should be noted. First, the sample was relatively small in size and consisted solely of friend pairs recruited from a single women’s college in Japan, limiting generalisability to other genders, institutions, and cultural contexts. Cultural and educational variability in friendship processes across Asian populations has been reported [55], and future studies should examine larger and more diverse samples, including male participants. Second, although no statistically significant differences were observed between the friend-pair and random-pair groups, the analyses did not control for potential third variables such as friendship duration, socio-economic background, or shared environments. One might consider that, because participants in the friend-pair group were drawn from the same institution, the observed similarity may reflect homophily based on shared background characteristics rather than friendship per se. Conversely, participants in the random-pair group were university students recruited from outside the institution and may therefore have been more heterogeneous, which in turn could have contributed to larger absolute differences, independent of any effect of friendship. To better clarify the role of sample heterogeneity, future research should compare friend pairs with randomly formed pairs drawn from the same institution, thereby controlling for heterogeneity in the random-pair group. Third, although the study focused on dyadic similarity, it did not employ specialised dyadic or multilevel statistical models, which are recommended for addressing interdependence in dyadic data [56]. Fourth, the present study relied on cross-sectional and correlational data, making it difficult to draw causal conclusions. Fifth, methodological constraints—including different data collection modes across groups and the use of a single-item measure of subjective similarity—may have influenced how participants responded and reduced reliability. Future research would benefit from multi-item measures of perceived similarity and procedures intended to be equivalent across groups. Despite these limitations, the present study provides preliminary evidence suggesting that similarity in Extraversion and in moral attitudes toward manners is associated with perceived personality similarity among Japanese young women. 

## Supplementary Information

Below is the link to the electronic supplementary material.

Supplementary Material 1.

## Data Availability

The datasets generated and/or analysed during the present study are available in the OSF repository at https://osf.io/v4xg5/.

## Abbreviations

M: Mean
Mdn: Median
SD: Standard deviation
