# Supplementary material for: An exploratory study of perceived and actual similarity in extraversion and moral attitudes toward manners in young adult friendships: Evidence from Japanese female students
Source: BMC Res Notes. 2026 Apr 10;19:230. doi: 10.1186/s13104-026-07810-w (PMC13185362; doi:10.1186/s13104-026-07810-w)
Supplement: Supplementary file 1 — Supplementary Material 1. [file 13104_2026_7810_MOESM1_ESM.docx]

Supplementary Information

**An exploratory study of perceived and actual similarity in extraversion and moral attitudes toward manners in young adult friendships: Evidence from Japanese female students**

Hiroshi Nitta^1, 2*^

^1^ Graduate School of Letters, Kyoto University, Yoshida Honmachi Sakyo-ku, Kyoto-shi, Kyoto, 606-8501, Japan

^2^ Graduate School of Human-Environment Studies, 744 Motooka, Nishi-ku, Fukuoka-shi, Fukuoka, 819-0395, Japan

^*^Correspondence concerning this article should be addressed to:

Hiroshi Nitta, Graduate School of Letters, Kyoto University, Yoshida Honmachi Sakyo-ku, Kyoto-shi, Kyoto, 606-8501, Japan

Tel & Fax: +81-75-753-2693

Email: h.nitta2030@gmail.com

This supplementary information includes:

1. Items of questionnaires used in the present study
2. Cronbach’s alpha coefficient of each variable in the friend-pair and random-pair groups
3. Table of permutation tests examining group differences across variables
4. Discussion on non-significant group differences across variables between the friend-pair and random-pair groups
5. Discussion on non-significant associations between subjective and objective similarity across variables in the friend-pair group
6. Tables reporting supplementary correlational analyses of (1) associations among personality traits, moral attitudes, and conformity in the friend-pair and random-pair groups, and (2) associations between subjective and objective similarity for these variables in the friend-pair group
7. **Items of questionnaires used in the present study**
   1. ***Scale of personality scale***

A short form of the Japanese Big Five personality traits [1] comprises 29 items across five subscales: (1) Extraversion (Items 1, 5, 10, 15, and 21), (2) Agreeableness (Items 4, 9, 14, 17, 19, and 26), (3) Conscientiousness (Items 3, 8, 13, 16, 20, 23, and 28), (4) Neuroticism (Items 6, 11, 18, 27, and 29), and (5) Openness to Experience (Items 2, 7, 12, 22, 24, and 25). Of the 29 items, 9 were reverse-coded (Items 3, 5, 8, 9, 13, 14, 16, 23, and 26).

1. Talkative

2. Imaginative

3. Careless

4. Gentle

5. Quiet

6. Easily anxious

7. Versatile

8. Disorganized

9. Quick-tempered

10. Cheerful

11. Worry-prone

12. Open-minded

13. Lazy

14. Irritable

15. Outgoing

16. Go-with-the-flow

17. Tolerant

18. Easily discouraged

19. Kind

20. Organized

21. Sociable

22. Quick-witted

23. Impulsive

24. Broad interests

25. Curious

26. Self-centred

27. Easily tense

28. Meticulous

29. Depressive

- 1. ***Scale of moral attitudes***

A Japanese questionnaire measuring moral and legal consciousness [2] comprises 15 items across three subscales: Legal violations, Ordinance violations, and Violations of manners. Each of the 15 items, five of which measure the level of tolerance for behaviours related to the three subscales.

**1.2.1. Legal violations**

1. Riding a train or bus without paying the fare
2. Driving a car under the influence of alcohol
3. Offering alcohol to minors
4. Parking a car for an extended period in a no-parking zone
5. Shoplifting

**1.2.2. Ordinance violations**

1. Taking photos of other passengers on a train without their permission

2. Littering empty cans on the street

3. Leaving dog faeces in public places

4. Vandalizing public toilets with graffiti

5. Posting malicious comments about others on the internet

**1.2.3. Violations of manners**

1. Playing loud music at night that can be heard by neighbours

2. Speaking loudly in a library without concern

3. Diving into a crowded swimming pool

4. Putting one’s towel into the bathtub at a public bath

5. Cutting in line where people are waiting

- 1. ***Scale of conformity***

The Japanese version of the conformity scale was developed by Yokota and Nakanishi [3] and comprises 23 items, with informational influence-based conformity (“informational conformity”) consisting of 10 items and normative influence-based conformity (“normative conformity”) consisting of 13 items. Specifically, informational conformity includes items 2, 10, 12, 14, 16, 17, 18, 19, 20, and 23 whereas normative conformity includes items 1, 3, 4, 5, 6, 7, 8, 9, 11, 13, 15, 21, and 22. Of the 23 items, 2 were reverse-coded (Items 18 and 21).

1. If insisting on my own opinion would disrupt the atmosphere, I feel more comfortable saying nothing.
2. I feel very relieved when my opinion matches that of others.
3. I often agree with others, even if it's not how I truly feel, because I worry about how people will react.
4. I care more about others’ judgments than my own thoughts.
5. Even when I don't agree, I often go along with others reluctantly.
6. When I do something with a friend, they usually make the decisions.
7. I feel awkward if I'm the only one in a group with a different opinion.
8. If someone’s opinion is highly persuasive, I change my own view and cooperate with them.
9. I often adjust myself to others to avoid disrupting the group.
10. When choosing classes, I decide based on information I get from others about the course.
11. I agree with the group even if it goes against what I believe.
12. I might wear trendy clothes even if they aren’t my personal preference.
13. I tend to follow the standards of the group.
14. When eating out, I refer to magazines or reviews for guidance.
15. Sometimes I feel like I can't fully express myself in a group.
16. If people around me are crossing the street on a red light, I think it must be safe to cross too.
17. When choosing a movie to watch, I rely on the opinions of people who’ve already seen it.
18. I’d rather be independent than follow a group.
19. Group consensus is often more accurate than an individual’s opinion.
20. My political decisions, such as in elections, are influenced by my parents' opinions.
21. Regardless of what others think, I tend to stick to my own beliefs.
22. I sometimes lose confidence in my own judgment and refer to others’ opinions.
23. When I have to make an important decision quickly, I observe others' actions to confirm my own judgment.
    1. ***Subjective similarity with your friend***

For the following items, how similar do you think you and the friend who participated with you this time are ? (1 = “Not similar at all” to 5 = “Very similar”)

1. Personality
2. Mindset
3. **Cronbach’s alpha coefficient of each variable in the friend-pair and random-pair groups**

***2.1. Personality***

The internal consistency (Cronbach’s alpha) of the Big Five traits was as follows for the friend-pair and random-pair groups, respectively: Extraversion (Friend: 0.79, Random: 0.75); Agreeableness (Friend: 0.77, Random: 0.70); Conscientiousness (Friend: 0.69, Random: 0.75); Neuroticism (Friend: 0.85, Random: 0.82); and Openness to Experience (Friend: 0.79, Random: 0.64).

***2.2. Moral attitudes***

The internal consistency (Cronbach’s alpha) of moral attitudes was as follows for the friend-pair and random-pair groups, respectively: Legal violations (Friend: 0.74, Random: 0.84); Ordinance violations (Friend: 0.81, Random: 0.84); and Violations of manners (Friend: 0.69, Random: 0.86).

***2.3. Conformity***

The internal consistency (Cronbach’s alpha) of conformity was as follows for the friend-pair and random-pair groups, respectively: Informational conformity (Friend: 0.65, Random: 0.70) and Normative conformity (Friend: 0.89, Random: 0.87).

1. **Table of permutation tests examining group differences across variables**

The table below reports permutation test results examining group differences between the friend-pair and random-pair groups across variables (Table S1).

Table S1. Results of permutation tests comparing friend and random pairs in mean absolute differences across variables (*N* = 30 dyads per group)

|  | **Variable** |  | **Observed Mean** | **Permutation Mean** |  | ***p*** |  | **CI_lower_** | **CI_upper_** |
| --- | --- | --- | --- | --- | --- | --- | --- | --- | --- |
| ***Big Five personality***  ***traits*** | **Extraversion** |  | 1.23 | 1.13 |  | 0.807 |  | 0.90 | 1.34 |
|  | **Agreeableness** |  | 1.14 | 0.99 |  | 0.948 |  | 0.79 | 1.18 |
|  | **Conscientiousness** |  | 1.01 | 0.98 |  | 0.649 |  | 0.77 | 1.16 |
|  | **Neuroticism** |  | 1.45 | 1.41 |  | 0.578 |  | 1.11 | 1.66 |
|  | **Openness to Experience** |  | 1.02 | 1.02 |  | 0.494 |  | 0.81 | 1.22 |
| ***Moral***  ***attitudes*** | **Legal** |  | 0.51 | 0.59 |  | 0.125 |  | 0.46 | 0.71 |
|  | **Ordinance** |  | 0.49 | 0.52 |  | 0.240 |  | 0.41 | 0.62 |
|  | **Manner** |  | 0.63 | 0.59 |  | 0.751 |  | 0.47 | 0.69 |
| ***Conformity*** | **Informational** |  | 0.52 | 0.60 |  | 0.091 |  | 0.48 | 0.71 |
|  | **Normative** |  | 0.75 | 0.81 |  | 0.227 |  | 0.65 | 0.97 |

Note. The absolute differences were obtained by computing the absolute value of the score difference between the two individuals in each dyad (i.e., |score₁ − score₂|), where smaller values represented higher within-dyad similarity. Observed Mean denotes the mean of the observed score differences for each variable. Permutation Mean indicates the average score difference obtained from the permutation distribution. *P*-values denotes the two-tailed permutation *p*-value (10,000 iterations). CI_lower_ and CI_upper_ indicate the 2.5th and 97.5th percentiles of the permutation distribution, respectively.

1. **Discussion on non-significant group differences across variables between the friend-pair and random-pair groups**

This section provides supplementary discussion on the non-significant differences in similarity in personality traits, moral attitudes, and conformity in each variable between

the friend-pair and random-pair groups.

First, non-significant group differences were found for personality traits between the friend-pair and random-pair groups. Based on previous findings, I expected that personality traits would be more similar between friend pairs than between randomly formed pairs. As one possible interpretation, it is plausible that the absence of similarity in personality traits among friends reflects the inherently individual and relatively stable nature of personality. Personality traits are largely shaped by enduring dispositions and genetic factors, and thus are less likely to be affected by day-to-day interpersonal dynamics [4, 5]. Consequently, even close friends may not exhibit strong personality resemblance, as such traits remain relatively stable and independent across individuals. Moreover, previous studies suggest that friendship quality is driven more by perceived similarity than by objective personality resemblance [6].

Second, similarly, there were non-significant group differences in moral attitude similarity. The finding was inconsistent with the previous findings showing that friends tend to resemble one another in moral attitudes [7]. An alternative interpretation of the present findings is that attitudes towards legal, ordinance, and manner violations were shared as group norms across participants, resulting in no significant group differences.

Although the friend-pair and random-pair groups differed in the presence or absence of a friendship relationship, participants shared multiple higher-level characteristics across dyads, including age, gender, country of residence, and student status. These shared characteristics may have contributed to broadly similar moral attitudes across the two groups. In addition to shared characteristics, in cultural contexts that emphasise norm adherence and interdependence, such as Japan, social norms may become moralised and broadly shared beyond specific friendship ties [8–10]. Consequently, non-significant group differences in moral attitudes may reflect moral attitudes shared at a broader demographic and cultural level rather than differences between friend and random pairs.

Finally, I examined whether the friend-pair group would be more similar in conformity than the random-pair group; however, no significant group differences were found. It is plausible that the absence of similarity in conformity among friends reflects the fact that conformity is shaped strongly by situational contexts rather than by enduring personal tendencies. Conformity tendencies are highly context-dependent and display low temporal stability. Conformity behaviour varies considerably depending on situational factors such as group norms, power dynamics, and social pressure at a given moment [11, 12]. Therefore, conformity reflects a context-sensitive behavioural pattern that is unlikely to be shared consistently among friends as a fixed individual difference.

1. **Discussion on non-significant associations between subjective and objective similarity across variables in the friend-pair group**

This section provides supplementary discussion on the non-significant correlations between perceived and objective similarity in each variable among friend pairs.

Prior research indicates that Extraversion is among the most behaviourally observable of the Big Five traits, as it manifests in overt sociability, talkativeness, and activity level — features that enhance accurate recognition by others [13–15]. These observable cues may also be associated with perceptions of similarity when individuals infer personality characteristics from social behaviour.

By contrast, other traits (e.g., Agreeableness; Conscientiousness; Neuroticism), that are less directly observable, less behaviourally distinct, or expressed privately, may not be reliably associated with judgments of subjective similarity. Evidence showed that traits that are difficult to observe typically yield weak interpersonal accuracy [13, 16, 17]. This reduces the likelihood that perceived similarity aligns with objective similarity.

One possible explanation for why only manners-related moral attitudes correlated with perceived similarity may be explained by differences in the social and interactional nature of moral domains. Moral psychology distinguishes multiple types of moral concerns, and not all moral domains are equally social or interaction-based [18, 19]. Violations of manners can be viewed as breaches of “everyday norms”, that is, the unwritten rules that specify the appropriateness of behaviour in ordinary social situations across societies [20]. In this sense, manners constitute a conventional form of interpersonal norm that governs everyday interaction, and violations of manners represent deviations from such norms. These norms develop through repeated joint experiences in everyday interactional contexts. Because friendships are characterised by extensive shared routines and environments, convergence is more likely to occur in these interpersonal norms. This is less likely to occur in moral domains that are less frequently activated or jointly engaged in everyday life, such as concerns related to legality or ordinances. Consequently, friends may judge more accurately how similar they are to one another regarding this domain (i.e., manner-related attitude), producing the observed association. In contrast, legal and ordinance violations are influenced more by abstract values, cultural learning, and individual belief systems, which do not necessarily converge among friends [21]. This may explain why only manners-related moral attitudes showed evidence of subjective–objective correspondence. Moreover, conformity-related traits vary greatly across contexts and depend strongly on abstract principles rather than behaviours, making them harder to judge accurately [22].

Furthermore, perceived similarity in mindset did not correlate with any objective differences. In this study, mindset refers to a general way of thinking that shapes how individuals perceive and respond to various situations [23]. Mindsets function as broader meaning systems that organize goals, interpretations, and reactions across situations, rather than as specific personality traits [24], and friends may not accurately infer each other's mindset from everyday interactions. Taken together, these non-significant findings highlight that perceived similarity does not generalize broadly across traits but appears limited to specific, behaviourally salient domains.

1. **Tables reporting supplementary correlational analyses of (1) associations among personality traits, moral attitudes, and conformity in the friend-pair and random-pair groups, and (2) associations between subjective and objective similarity for these variables in the friend-pair group**

The tables below present supplementary correlational analyses of associations among personality traits, moral attitudes, and conformity in the friend-pair and random-pair groups, together with associations between subjective and objective similarity in the friend-pair group.

Table S2. Intercorrelations among all measured variables for each pair group (*N* = 30 dyads per group)

| **Group** | | **Variable** | | **1** | **2** | **3** | **4** | **5** | **6** | **7** | **8** | **9** | **10** |
| --- | --- | --- | --- | --- | --- | --- | --- | --- | --- | --- | --- | --- | --- |
| **Friend** | ***Big Five***  ***personality***  ***traits*** | | **1** | 1.00 | 0.44* | -0.21 | -0.47** | 0.51** | 0.25 | 0.01 | 0.12 | -0.31 | -0.52** |
|  |  |  | **2** |  | 1.00 | 0.47** | -0.25 | 0.38* | -0.12 | -0.22 | 0.07 | -0.22 | -0.17 |
|  |  |  | **3** |  |  | 1.00 | 0.25 | 0.06 | -0.25 | -0.19 | 0.06 | 0.01 | 0.09 |
|  |  |  | **4** |  |  |  | 1.00 | -0.46** | -0.19 | -0.09 | -0.26 | 0.43* | 0.64*** |
|  |  |  | **5** |  |  |  |  | 1.00 | 0.09 | 0.00 | 0.22 | -0.36 | -0.53** |
|  | ***Moral***  ***attitudes*** | | **6** |  |  |  |  |  | 1.00 | 0.73*** | 0.75*** | -0.07 | -0.30 |
|  |  |  | **7** |  |  |  |  |  |  | 1.00 | 0.70*** | 0.12 | -0.22 |
|  |  |  | **8** |  |  |  |  |  |  |  | 1.00 | -0.09 | -0.28 |
|  | ***Conformity*** | | **9** |  |  |  |  |  |  |  |  | 1.00 | 0.53** |
|  |  |  | **10** |  |  |  |  |  |  |  |  |  | 1.00 |
| **Random** | ***Big Five***  ***personality***  ***traits*** | | **1** | 1.00 | 0.04 | -0.10 | -0.26 | 0.25 | 0.08 | 0.08 | 0.08 | 0.16 | 0.36 |
|  |  |  | **2** |  | 1.00 | -0.16 | -0.44* | 0.25 | 0.25 | 0.32 | 0.17 | -0.22 | -0.21 |
|  |  |  | **3** |  |  | 1.00 | 0.23 | -0.35 | -0.04 | 0.06 | 0.17 | -0.06 | -0.11 |
|  |  |  | **4** |  |  |  | 1.00 | -0.30 | -0.12 | -0.27 | -0.23 | -0.16 | 0.04 |
|  |  |  | **5** |  |  |  |  | 1.00 | 0.08 | 0.16 | 0.06 | -0.12 | 0.26 |
|  | ***Moral***  ***attitudes*** | | **6** |  |  |  |  |  | 1.00 | 0.72*** | 0.58*** | 0.00 | -0.22 |
|  |  |  | **7** |  |  |  |  |  |  | 1.00 | 0.83*** | 0.06 | -0.32 |
|  |  |  | **8** |  |  |  |  |  |  |  | 1.00 | 0.16 | -0.09 |
|  | ***Conformity*** | | **9** |  |  |  |  |  |  |  |  | 1.00 | 0.57*** |
|  |  |  | **10** |  |  |  |  |  |  |  |  |  | 1.00 |

Note. Numbers correspond to the following variables: 1 = Extraversion; 2 = Agreeableness; 3 = Conscientiousness; 4 = Neuroticism; 5 = Openness to Experience; 6 = Legal; 7 = Ordinance; 8 = Manner; 9 = Informational; 10 = Normative. Pearson’s *r* coefficients are shown. **p* < 0.05, ***p* < 0.01, ****p* < 0.001.

Table S3. Within-dyad correlations for each variable in each pair group (*N* = 30 dyads per group)

| **Variable** | |  | **Friend group** | |  | **Random group** | |
| --- | --- | --- | --- | --- | --- | --- | --- |
|  |  |  | ***r*** | ***p*** |  | ***r*** | ***p*** |
| ***Big Five***  ***personality***  ***traits*** | **Extraversion** |  | -0.03 | 0.859 |  | -0.21 | 0.270 |
|  | **Agreeableness** |  | -0.31 | 0.092 |  | 0.19 | 0.326 |
|  | **Conscientiousness** |  | 0.04 | 0.837 |  | -0.08 | 0.664 |
|  | **Neuroticism** |  | 0.00 | 0.983 |  | -0.28 | 0.130 |
|  | **Openness to Experience** |  | -0.15 | 0.432 |  | 0.10 | 0.582 |
| ***Moral***  ***attitudes*** | **Legal** |  | 0.17 | 0.367 |  | -0.11 | 0.545 |
|  | **Ordinance** |  | 0.24 | 0.192 |  | -0.17 | 0.358 |
|  | **Manner** |  | -0.14 | 0.470 |  | -0.30 | 0.102 |
| ***Conformity*** | **Informational** |  | 0.23 | 0.231 |  | 0.28 | 0.128 |
|  | **Normative** |  | 0.29 | 0.120 |  | 0.05 | 0.802 |

Note. *r* represents Pearson’s product–moment correlation coefficient.

Table S4. Within-dyad correlations between subjective similarity in personality or mindset and each variable in the friend-pair group (*N* = 30 dyads)

|  |  |  | ***Subjective similarity*** | |
| --- | --- | --- | --- | --- |
|  |  |  | ***Personality*** | ***Mindset*** |
| ***Objective similarity*** | ***Big Five***  ***personality***  ***traits*** | **Extraversion** | 0.43* | 0.22 |
|  |  | **Agreeableness** | 0.57*** | 0.34 |
|  |  | **Conscientiousness** | 0.16 | -0.03 |
|  |  | **Neuroticism** | -0.13 | -0.35 |
|  |  | **Openness to Experience** | 0.17 | 0.19 |
|  | ***Moral***  ***attitudes*** | **Legal** | 0.04 | -0.14 |
|  |  | **Ordinance** | -0.13 | -0.20 |
|  |  | **Manner** | -0.11 | -0.29 |
|  | ***Conformity*** | **Informational** | -0.05 | -0.23 |
|  |  | **Normative** | -0.15 | -0.25 |

Note. Pearson’s *r* coefficients are shown. **p* < 0.05, ****p* < 0.001.

**References**

1. Tsutomu Namikawa, Iori Tani, Takafumi Wakita, Ryuichi Kumagai, Ai Nakane, Hiroyuki Noguchi. Development of a short form of the Japanese Big- Five Scale, and a test of its reliability and validity. The Japanese Journal of Psychology. 2012;2:91–9. https://doi.org/https://doi.org/10.4992/jjpsy.83.91.

2. Mizokawa A, Koyasu M. The influence of foreign experiences and critical thinking disposition on moral and legal consciousness. Japanese Journal of Psychology. 2020;90:562–71. https://doi.org/10.4992/JJPSY.90.18055.

3. Yokoi R, Nakayachi K. Trust in autonomous cars: The role of value similarity and capacity for sympathy. THE JAPANESE JOURNAL OF EXPERIMENTAL SOCIAL PSYCHOLOGY. 2021;61:22–7. https://doi.org/10.2130/jjesp.2020.

4. Vukasović T, Bratko D. Heritability of personality: A meta-analysis of behavior genetic studies. Psychol Bull. 2015;141:769–85. https://doi.org/10.1037/bul0000017.

5. McCrae RR, John OP. An Introduction to the Five‐Factor Model and Its Applications. J Pers. 1992;60:175–215. https://doi.org/10.1111/j.1467-6494.1992.tb00970.x.

6. Selfhout M, Burk W, Branje S, Denissen J, van Aken M, Meeus W. Emerging Late Adolescent Friendship Networks and Big Five Personality Traits: A Social Network Approach. J Pers. 2010;78:509–38. https://doi.org/10.1111/j.1467-6494.2010.00625.x.

7. Pozzoli T, Gini G. Friend similarity in attitudes toward bullying and sense of responsibility to intervene. Soc Influ. 2013;8:161–76. https://doi.org/10.1080/15534510.2012.716372.

8. Takamatsu R, Min MC, Wang L, Xu W, Taniguchi N, Takai J. Moralization of Japanese cultural norms among student sojourners in Japan. International Journal of Intercultural Relations. 2021;80:242–9. https://doi.org/10.1016/j.ijintrel.2020.12.001.

9. Mcpherson M, Smith-Lovin L, Cook JM. Birds of a Feather: Homophily in Social Networks. Annu Rev Sociol. 2001;27:415–44. https://doi.org/https://doi.org/10.1146/annurev.soc.27.1.415.

10. Rose Markus H, Kitayama S. Culture and the Self: Implications for Cognition, Emotion, and Motivation. 1991.

11. Bond R, Smith PB. Culture and conformity: A meta-analysis of studies using Asch’s (1952b, 1956) line judgment task. Psychol Bull. 1996;119:111–37. https://doi.org/10.1037/0033-2909.119.1.111.

12. Cialdini RB, Goldstein NJ. Social influence: Compliance and conformity. Annu Rev Psychol. 2004;55:591–621. https://doi.org/10.1146/annurev.psych.55.090902.142015.

13. Connelly BS, Ones DS. An other perspective on personality: Meta-analytic integration of observers’ accuracy and predictive validity. Psychol Bull. 2010;136:1092–122. https://doi.org/10.1037/a0021212.

14. Vazire S. Who Knows What About a Person? The Self-Other Knowledge Asymmetry (SOKA) Model. J Pers Soc Psychol. 2010;98:281–300. https://doi.org/10.1037/a0017908.

15. Back MD, Schmukle SC, Egloff B. A closer look at first sight: Social relations lens model analysis of personality and interpersonal attraction at zero acquaintance. Eur J Pers. 2011;25:225–38. https://doi.org/10.1002/per.790.

16. Funder DC. On the Accuracy of Personality Judgment: A Realistic Approach. 1995.

17. Letzring TD. The good judge of personality: Characteristics, behaviors, and observer accuracy. J Res Pers. 2008;42:914–32. https://doi.org/10.1016/j.jrp.2007.12.003.

18. Graham J, Haidt J, Koleva S, Motyl M, Iyer R, Wojcik SP, et al. Moral Foundations Theory: The Pragmatic Validity of Moral Pluralism. In: Advances in Experimental Social Psychology. Academic Press Inc.; 2013. p. 55–130. https://doi.org/10.1016/B978-0-12-407236-7.00002-4.

19. Haidt J. The emotional dog and its rational tail: A social intuitionist approach to moral judgment. Psychol Rev. 2001;108:814–34. https://doi.org/10.1037/0033-295X.108.4.814.

20. Eriksson K, Strimling P, Vartanova I, Simpson B, Persson M, Abdi KA, et al. Everyday norms have become more permissive over time and vary across cultures. Communications Psychology. 2025;3. https://doi.org/10.1038/s44271-025-00324-4.

21. Feldman S. Values, ideology, and the structure of political attitudes. In: Sears DO, Huddy L, Jervis R, editors. Oxford handbook of political psychology. Oxford University Press; 2003. p. 477–508.

22. Goodwin GP. Moral Character in Person Perception. Curr Dir Psychol Sci. 2015;24:38–44. https://doi.org/10.1177/0963721414550709.

23. Doss K, Bloom L. Mindset and the desire for feedback during creative tasks. Journal of Creativity. 2023;33. https://doi.org/10.1016/j.yjoc.2023.100047.

24. Molden DC, Dweck CS. Finding “Meaning” in Psychology: A Lay Theories Approach to Self-Regulation, Social Perception, and Social Development. American Psychologist. 2006;61:192–203. https://doi.org/10.1037/0003-066X.61.3.192.
